# Sildenafil Therapy Normalizes the Aberrant Metabolomic Profile in the Comt^−/−^ Mouse Model of Preeclampsia/Fetal Growth Restriction

**DOI:** 10.1038/srep18241

**Published:** 2015-12-15

**Authors:** Joanna L. Stanley, Karolina Sulek, Irene J. Andersson, Sandra T. Davidge, Louise C. Kenny, Colin P. Sibley, Rupasri Mandal, David S. Wishart, David I. Broadhurst, Philip N. Baker

**Affiliations:** 1 Liggins Institute, University of Auckland, New Zealand; 2 Department of Medicine, University of Alberta, Edmonton, Canada; 3 Women and Children’s Health Research Institute, University of Alberta, Edmonton, Canada; 4 Anu Research Centre, University College Cork, Ireland; 5 Maternal & Fetal Health Research Centre, Institute of Human Development, Faculty of Medical and Human Sciences, University of Manchester, UK; 6 The Metabolomics Innovation Centre (TMIC), University of Alberta, Edmonton, Canada

## Abstract

Preeclampsia (PE) and fetal growth restriction (FGR) are serious complications of pregnancy, associated with greatly increased risk of maternal and perinatal morbidity and mortality. These complications are difficult to diagnose and no curative treatments are available. We hypothesized that the metabolomic signature of two models of disease, catechol-O-methyl transferase (COMT^−/−^) and endothelial nitric oxide synthase (Nos3^−/−^) knockout mice, would be significantly different from control C57BL/6J mice. Further, we hypothesised that any differences in COMT^−/−^ mice would be resolved following treatment with Sildenafil, a treatment which rescues fetal growth. Targeted, quantitative comparisons of serum metabolic profiles of pregnant Nos3^−/−^, COMT^−/−^ and C57BL/6J mice were made using a kit from BIOCRATES. Significant differences in 4 metabolites were observed between Nos3^−/−^ and C57BL/6J mice (p < 0.05) and in 18 metabolites between C57BL/6J and COMT^−/−^ mice (p < 0.05). Following treatment with Sildenafil, only 5 of the 18 previously identified differences in metabolites (p < 0.05) remained in COMT^−/−^ mice. Metabolomic profiling of mouse models is possible, producing signatures that are clearly different from control animals. A potential new treatment, Sildenafil, is able to normalize the aberrant metabolomic profile in COMT^−/−^ mice; as this treatment moves into clinical trials, this information may assist in assessing possible mechanisms of action.

Preeclampsia (PE) and fetal growth restriction (FGR) are complications of pregnancy, which together affect around 10% of pregnancies worldwide^1^,^2^. PE is characterized by the onset of hypertension and proteinuria after the 20^th^ week of gestation, whilst FGR (which may or may not be associated with PE) is defined as a fetus that fails to reach its genetic growth potential. Together, these complications are a leading cause of maternal and perinatal morbidity and mortality^3^,^4^,^5^. Additionally, lower birth weight is associated with an increased risk of developing diseases in later life, including cardiac and metabolic diseases^6^,^7^. Despite the massive health and societal costs of these conditions, treatment options for PE and FGR are limited and often involve premature delivery of the fetus, which itself carries significant risk to both mother and child.

The development of new therapeutic agents for these complications of pregnancy has been significantly impaired by both a limited ability to diagnose PE/FGR, as well as a lack of suitable animal models with which to test new treatments. The etiology of these conditions is complex, and likely multi-factorial, although increased uterine artery resistance is commonly observed in both cases^8^. An exciting recent development has been the identification of genetically-modified mouse models which display characteristics of PE/FGR. Two of these models are of particular interest, namely the endothelial nitric oxide synthase knockout (Nos3^−/−^) mouse and the catechol-O-methyl transferase knockout (COMT^−/−^) mouse. Nitric oxide (NO) has been observed to play a significant role in pregnancy, specifically by mediating some of the maternal cardiovascular adaptations necessary to ensure adequate placental perfusion^9^,^10^ and maintenance of normal maternal blood pressure^11^. Unsurprisingly, therefore, investigation of the Nos3^−/−^ mouse during pregnancy has highlighted significant abnormalities when compared with wild-type counterparts. Key observations, which recapitulate some of the clinical signs of disease, include abnormal uterine artery remodeling^12^ and uterine artery dysfunction^13^, increased placental hypoxia^14^,^15^ and FGR^13^,^15^.

Catechol-O-methyl transferase (COMT) is one of the enzymes involved in the metabolism of 17β-estradiol to 2- and 4-methoxyestradiol^16^. Studies of the COMT^−/−^ mouse during pregnancy have demonstrated maternal hypertension and increased proteinuria at late gestation^17^, as well as aberrant umbilical artery Doppler waveforms and FGR^18^; again demonstrating the ability of a genetically-modified mouse model to recapitulate some of the key clinical signs of disease.

Given the multifactorial etiology of PE and FGR, there are advantages to studying multiple animal models of disease with differing pathophysiological mechanisms. The aim of the study detailed here was to analyze the metabolomic signature of two mouse models of PE/FGR (COMT^−/−^ and Nos3^−/−^ mice), as well as C57BL/6J control mice. We hypothesized that differences in the metabolomic profiles of COMT^−/−^ and Nos3^−/−^ mice, in comparison with control mice, would be demonstrated.

The development of appropriate animal models has allowed for the testing of potential new treatments. One such treatment identified as having potential therapeutic benefits is Sildenafil citrate; it is hypothesized that, given its vasodilatory effects, Sildenafil citrate therapy would reduce uterine artery resistance and enhance uteroplacental perfusion. A small study of women identified as having severe early-onset FGR were treated with Sildenafil citrate; this study demonstrated increased fetal abdominal circumference growth compared with untreated controls^19^. It has also been observed that Sildenafil citrate treatment normalizes umbilical artery Doppler waveforms and improves fetal growth in the COMT^−/−^ mouse model^18^. Although positive effects of Sildenafil citrate treatment have been demonstrated, it is important to fully understand its mechanisms of action of to ensure it is used in a safe and beneficial manner. Given its positive biological effects in the COMT^−/−^ mouse model, we further hypothesized that differences in the metabolomic profile of COMT^−/−^ mice would be at least partially resolved following treatment with Sildenafil citrate; the metabolomic profiles of COMT^−/−^ and control mice treated with Sildenafil citrate were thus assessed.

The results of such analyses could thus provide further insight into the pathophysiological mechanisms of these models and help understand the mode of action of potential therapies.

## Results

Across all groups of mice studied, a total of 149 metabolites were identified to MSI level 1 and quantified (see Supplementary Table S1 online for full list of identified metabolites). The metabolites identified belonged to a number of different classes, including acyl carnitines, glycerophospholipids, sphingolipids, amino acids and biogenic amines such as creatinine and kyneurenine.

### Global changes in the metabolomic signature

**Figure 1:**
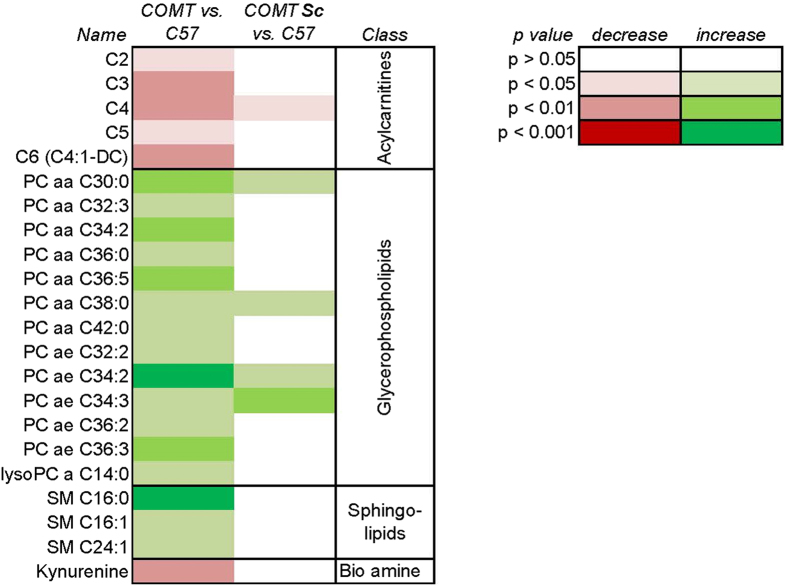
COMT^−/−^ mice demonstrated significant differences in their serum metabolomic signature compared with control C57BL/6J mice; these were largely resolved by Sildenafil citrate treatment. Heat map representing statistical differences, as assessed by Kruskal-Wallis test, in metabolite serum concentrations in either COMT^−/−^ vs. control C57BL/6 J mice or COMT^−/−^ mice treated with Sildenafil citrate (Sc) vs. control C57BL/6 J mice. Legend shows color-coding for statistical differences in the concentrations of given metabolites.

Initially, the metabolomic signature of control C57BL/6J mice was compared with that of Nos3^−/−^ and COMT^−/−^ mice. 4 metabolites were significantly altered in the serum of Nos3^−/−^ compared with C57BL/6J mice (see Supplementary Figure S1 online); serum concentrations of carnitine (p < 0.05), lysophosphatidylcholine acyl C26:0 (p < 0.01), carnosine (p < 0.05) and creatinine (p < 0.05) were all significantly increased compared with C57BL/6J mice. In comparison, a total of 18 metabolites were significantly altered in COMT^−/−^ compared with C57BL/6J mice (p < 0.05; Fig. 1, Table 1); concentrations of numerous glycerophospholipids and sphingolipids were increased in COMT^−/−^ mice, whilst concentrations of kyneurenine, and a number of acyl carnitines were decreased.

**Figure 2:**
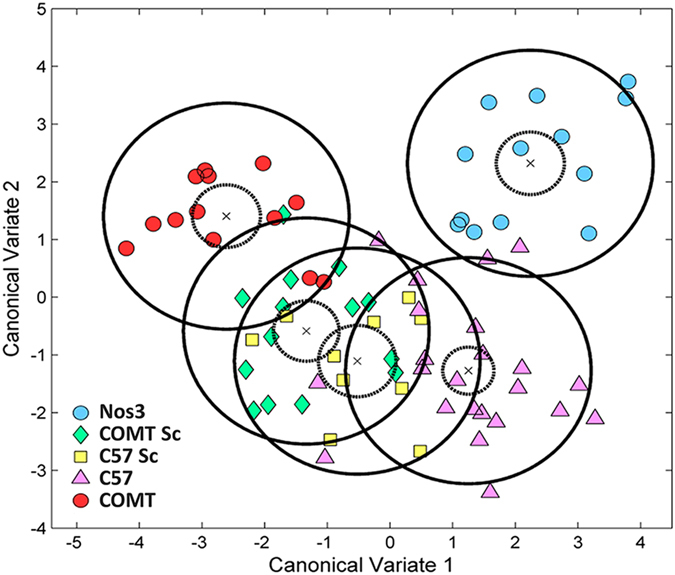
Significant multivariate mean differences were observed in the metabolomic signature between two mouse models of PE/FGR with differing underlying pathologies. Canonical Variate Analysis (CVA) was performed using those metabolites whose serum concentrations were significantly changed (identified in Figure S1, p < 0.05). Data are presented as CV1 vs. CV2 of Nos3^−/−^, COMT^−/−^, C57BL/6J mice, where Sc indicates those mice that were treated with Sildenafil citrate. Analysis demonstrated significant differences in the metabolomic signature between Nos3^−/−^ and COMT^−/−^ mice, as well as between control mice. There was no significant difference between the profiles of COMT^−/−^ and C57BL/6 J mice which had been treated with Sildenafil citrate. Solid circles = 95% confidence intervals for each group population; dashed circles = 95% confidence intervals for the mean of each group; black cross = the mean of each group.

Canonical Variate Analysis (CVA) was then performed on metabolites that demonstrated significant differences in serum concentration. A scatter plot of the first and second canonical variates (Fig. 2) describes clear significant multivariate mean differences between different untreated mouse models.

### Effect of Sildenafil citrate treatment on the metabolomic profile

The metabolomic signature of COMT^−/−^ mice treated with Sildenafil citrate was initially compared with that of untreated C57BL/6J control mice. Differences in only 5 of the 18 significantly altered metabolites persisted (Fig. 1, Table 1).

CVA analysis further shows that both C57BL/6J and COMT^−/−^ mice treated with Sildenafil were not significantly different; further, there is a significant shift in COMT^−/−^ mice treated with Sildenafil toward the untreated/treated C57BL/6J mice clusters (Fig. 2).

**Figure 3:**
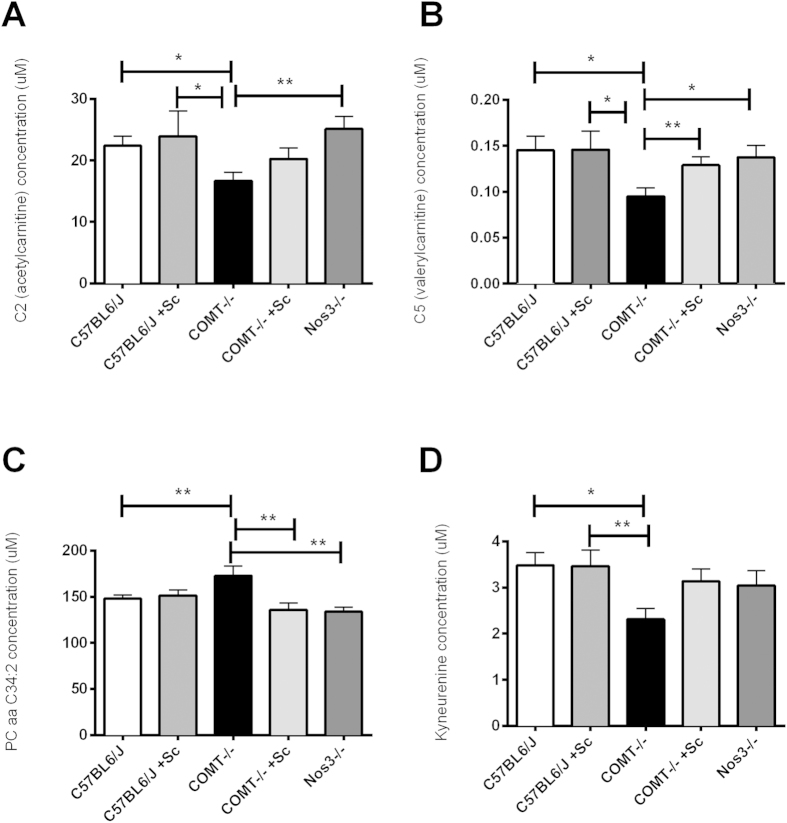
Sildenafil citrate treatment normalized changes in serum metabolite concentration in COMT^−/−^ mice. There was a significant difference in the serum concentration of metabolites belonging to a number of classes when samples from C57BL/6 J and COMT^−/−^ mice were compared. The metabolites which were significantly altered included acylcarnitines such as (**A)** C2 (acetylcarnitine) and (**B)** C5 (valerylcarnitine), glycerophospholipids such as (**C)** PC aa C34:2 (phosphatidylcholine diacyl C34:2), and (**D)** the bioamine kynurenine. These differences were not significant for the majority of metabolites when samples from C57BL/6 J mice and COMT^−/−^ mice treated with Sildenafil citrate were compared. n = 10–23, Kruskal-Wallis test; *p < 0.05, **p < 0.01. ‘Sc’ indicates mice were treated with Sildenafil citrate.

When specific changes in individual metabolites were examined, it was observed that the concentrations of acetylcarnitine (Fig. 3A), valerylcarnitine (Fig. 3B) and kynurenine (Fig. 3D) were significantly reduced in COMT^−/−^ mice compared with controls (p < 0.05). In comparison, the concentration of phosphatidylcholine diacyl C34:2 (Fig. 3C; p < 0.01) was significantly increased in COMT^−/−^ mice when compared with C57BL/6J mice. There were no differences in the serum concentrations of these metabolites between C57BL/6J mice and COMT^−/−^ mice treated with Sildenafil citrate (Fig. 3A–D; p > 0.05).

## Discussion

The etiology of both PE and FGR is complex and likely multifactorial. This is reflected in both the disparity of risk factors for the two conditions, as well as the difficulty in either diagnosing the conditions or developing potential new treatments. In order to investigate the possible pathophysiological mechanisms involved, and to test potential new treatments, we used two mouse models, with different genetic modifications, which have previously been reported to exhibit different signs of the two conditions. The underlying hypothesis linking the two models described here is a reduction in uteroplacental perfusion, thereby reflecting a key clinical indicator of disease^8^,^20^. The metabolomic signatures of the mouse models described here show significant differences compared with control mice.

A significant hurdle in the development of new treatments has been the lack of appropriate models with which to test their efficacy. Animals do not develop PE *per* se, although a number of models do now exist which exhibit different signs of the condition. The reduced uterine perfusion pressure (RUPP) model in rats, for instance, demonstrates increased blood pressure and proteinuria along with systemic maternal endothelial dysfunction, all of which are characteristic of PE^21^,^22^,^23^. Due to the permanent presence of mechanical vascular occlusion, however, it is difficult to assess the efficacy of treatments in this model, particularly if those treatments are targeted at improving utero-placental perfusion. Similar difficulties have been encountered in trying to appropriately model FGR. For instance, numerous investigators have utilized a maternal under-nutrition approach^24^,^25^. This does indeed result in FGR, but does not address the most important clinical cause, namely placental insufficiency. The two models used in the current study were chosen as they have been shown to demonstrate signs of PE and/or FGR, and display the key clinical characteristic of reduced uteroplacental perfusion.

The Nos3^−/−^ mouse contains a null mutation of the enzyme endothelial nitric oxide synthase (Nos3) gene^26^. Nos3 is one of 3 isoforms which catalyze the conversion of arginine to citrulline, producing nitric oxide (NO) as a by-product. In non-pregnant animals, the Nos3^−/−^ mouse exhibits an abnormal cardiovascular phenotype, including mild hypertension^27^ and endothelial dysfunction^28^. During pregnancy, NO plays a crucial role in mediating the important cardiovascular adaptations necessary for a successful pregnancy. It is therefore not surprising that the Nos3^−/−^ mouse demonstrates a number of changes in its adaptations to pregnancy when compared with C57BL/6J control mice. For instance, the pregnant dams are hypertensive, both before and during pregnancy^26^, and show a significantly reduced cardiac output^29^. There is a failure of the uterine arteries to remodel properly, leading to a reduced uterine artery diameter^12^. This has a significant impact on uteroplacental blood flow, and markers of placental hypoxia in the junctional zone are elevated in late gestation^30^. Significant impairment of placental nutrient transport has also been observed^13^, along with a consistent growth restriction phenotype^13^,^14^,^15^,^31^. Despite the significant and varied changes in both maternal physiology and placental function of Nos3^−/−^ mice, surprisingly only a small number of metabolites were significantly altered when the serum metabolic profile was compared to that of the C57BL/6J control mouse (supplementary Figure S1 online). Two of the metabolites whose serum concentrations were significantly increased, carnitine and carnosine, are known to exhibit antioxidant activity^32^,^33^. Their production may therefore have been increased in response to the increased levels of oxidative stress observed in pregnant Nos3^−/−^ mice^15^. It was interesting to note, however, that serum arginine concentrations were not affected by modulation of the Nos3 gene. This could suggest that not all metabolic alterations related to pregnancy disorders are reflected in the maternal serum. Some physiological changes might occur only in the specific tissue, without metabolic biomarkers visible in the blood specimen. Therefore, when searching for disease mechanisms and biological indicators, if possible, different types of biological materials should be taken into consideration.

The second model utilized in this study was the COMT^−/−^ mouse. Catechol-O-methyl transferase (COMT) converts, amongst other substrates, 17β-estradiol to 2-methoxyestradiol (2-ME). This model was firstly developed by Gogos and colleagues and has been widely used as a model of psychiatric disorders^34^, with its use in pregnancy being a relatively recent development. Human studies have proposed a potential role for COMT and 2-ME in PE; it has been observed that levels of both COMT and 2-ME are elevated during the third trimester of normal human pregnancy^35^, but are significantly lower in those women with severe PE^17^. Subsequently, numerous studies have observed signs of both PE and FGR in the COMT^−/−^ mouse model. These include late gestational hypertension, proteinuria and glomerular endotheliosis^17^ as well as abnormal umbilical artery Doppler waveforms and FGR^18^. Despite phenotypic similarities between the two models used here i.e. hypertension and FGR, there are differences in the pathophysiological mechanisms underlying these observations. For instance, no abnormalities in uterine artery Doppler waveforms or function were observed in the COMT^−/−^ mice^18^. There is, however, evidence of increased placental resistance^18^ and an imbalance of angiogenic factors in the COMT^−/−^ mouse^17^, suggesting that reduced placental angiogenesis may be a key mechanism in this model. It is unsurprising, therefore, that the COMT^−/−^ mouse displayed a different serum metabolomic signature when compared with the Nos3^−/−^ mouse, as well as the C57BL/6J control mouse (Fig. 2).

A significant number of metabolites were affected by knockout of the COMT gene (Fig. 1). Those affected were mainly from the acylcarnitine and lipid classes. Acylcarnitines are intermediary metabolites produced during the process of fatty acid oxidation; this is a key source of energy in a number of different tissues, including vascular smooth muscle^36^. Fatty acid oxidation takes place within the mitochondria, and a significant change in the concentration of acylcarnitines may be indicative of altered mitochondrial function. Typically in disease states, such as diabetes/insulin resistance, increased concentrations of acylcarnitines have been observed^37^. These are indicative of an increased lipid load, or incomplete fatty acid oxidation^38^,^39^. In this study, however, a significant decrease in acylcarnitine concentration was observed in COMT^−/−^ compared with control mice. This significant decrease was noted in short-chain (C2), medium-chain (C6) and amino acid-derived (C3, C4 and C5) acylcarnitines. As acylcarnitine concentrations are a direct reflection of the rate of fatty acid and amino acid oxidation, this could indicate reduced activity in these energy-producing pathways.

The placenta is an extremely metabolically active tissue, and any alterations in energy production could have significant effects on its development or function. Given that placental dysfunction appears to be key in mediating the phenotype observed in COMT^−/−^ mice^17^,^18^, it is interesting to note that mitochondrial dysfunction appears to play a role in the pathophysiology of FGR. For instance, following genome-wide expression analysis, Madaleneau *et al*. (2015) determined a significant reduction in the expression of a number of genes in placental tissue obtained from cases of FGR compared with normal pregnancies^40^. The genes which demonstrated reduced expression affected three of the five complexes of the mitochondrial respiratory chain and so likely had a significant impact on metabolism and energy production. Further, other rodent models of FGR have noted mitochondrial abnormalities in placental tissue^41^. It should be noted that other investigators have noted an increase in placental mitochondrial DNA content in placentas from cases of FGR^42^; although there is evidence from these studies of disturbed mitochondrial function, it is clear that changes in mitochondrial biogenesis and activity, and their role in the pathogenesis of placental insufficiency is likely complex. Little is known about the purpose of efflux of acylcarnitines into extracellular compartments, but the reduced concentrations observed here could be indicative of reduced mitochondrial biogenesis or activity in placental tissue.

Previously, we have observed that treatment of COMT^−/−^ mice with Sildenafil citrate resulted in a normalization of umbilical artery Doppler waveforms and an increase in fetal growth^18^. Treatment of COMT^−/−^ mice with Sildenafil citrate also resulted in the normalization of the concentration of all but one of the acylcarnitines. There are numerous studies that have demonstrated the ability of Sildenafil treatment to increase mitochondrial biogenesis and activity in cell lines^43^ and various animal models^44^,^45^. To date, the potential effects of Sildenafil on placental mitochondrial biogenesis/function have not been investigated, but the data presented here suggests this may be one mechanism by which Sildenafil is able to improve placental insufficiency and warrants further investigation.

Kynurenine was also observed to be significantly reduced in samples from COMT^−/−^ compared with C57BL/6J mice. Kynurenine is a metabolite of the amino acid tryptophan, and used in the production of niacin. Interestingly, niacin has been associated for many years with cardiovascular health. While initial benefits of niacin were believed to be associated with improved lipid profiles^46^, more recent studies have also demonstrated improved endothelial function associated with increased dietary niacin intake^47^. A reduction in kynurenine observed in COMT^−/−^ mice in this study may be reflected in a reduction in niacin production, which in turn may contribute to altered plasma lipid profiles and/or impaired endothelial function. COMT^−/−^ mice demonstrate a significant increase in placental resistance, which is reduced subsequent to Sildenafil treatment. The increased placental resistance may be attributable to increased vasoconstriction in the uteroplacental circulation, which is then ameliorated by Sildenafil. It is not known if niacin is vasoactive in the uteroplacental circulation and therefore contributing to these changes in placental resistance; *in vitro* studies should be performed to investigate the importance of this compound in this particular vascular bed.

Finally, significant increases in the concentrations of sphingolipids and glycerophospholipids were observed in COMT^−/−^ mice; these compounds are major structural components of plasma lipoproteins and biological membranes^48^, with important roles in the regulation of membrane protein trafficking and cell signaling and function. The altered concentration of these lipids observed in this study may result from cell membrane damage and subsequent release, associated with impaired placental growth, development and function. Improved uteroplacental perfusion, following treatment with Sildenafil, may be responsible for a reduction in cell damage, and therefore reduced release of phospholipids. Sildenafil, however, may also have more direct actions. For instance, the metabolism of sphingolipids results in the production of ceramides, which are key mediators of apoptosis. It has been observed that in women with PE, a condition associated with increased placental apoptosis, the serum concentration of various ceramides are increased^49^. Given the increased concentration of sphingolipids observed in COMT^−/−^ mice compared with controls, it appears there may be significant alterations in lipid metabolism pathways, which could result in an increased production of ceramides. Interestingly, nitric oxide is able to prevent ceramide-mediated apoptosis in a cGMP-dependent manner^50^. Given the links between increased placental apoptosis and the increased risk of PE^51^/FGR^52^, and the decrease in sphingolipids noted in mice treated with Sildenafil, this presents a further intriguing potential mechanism by which Sildenafil may mediate its beneficial effects.

Interestingly, the metabolites found to be altered in this model are from similar classes to those observed to be altered in plasma and placental samples taken from women who went on to develop FGR^53^ or PE^54^. For instance, in studies of plasma from women, taken in early pregnancy prior to the onset of FGR, acylcarnitines, phospholipids and sphingolipids were among the metabolites identified in a discriminatory metabolomic signature^53^. These alterations were later confirmed in both plasma samples obtained at the time of disease (venous cord plasma sampled at birth) as well as in a rat model of FGR^53^. Discovery and validation case-control studies have also identified an altered metabolomic signature early in pregnancy which is associated with the later development of PE^54^. Of note, this altered signature included changes in the concentration of acylcarnitines and phospholipids, again as noted in the study described here.

Further, recent analysis of placental gene expression profiles observed that one of the pathways significantly altered in PE was that of lipid metabolism^55^. PE and FGR are two conditions with complex, multifactorial etiologies. The identification of common pathways and/or mechanisms, which are altered in both human samples as well as across different animal models, is an important step in furthering our understanding of the pathophysiology of disease as well as developing and optimizing therapeutic strategies.

This study is the first to apply metabolomics to mouse models of preeclampsia and fetal growth restriction. It has illustrated that metabolomic profiling of mouse models of PE and FGR is able to not only produce a phenotypic signature, which is clearly distinguishable from controls, but is also characteristic of underlying pathophysiological mechanisms. Further, the ability of Sildenafil citrate to increase fetal growth was reflected in alterations of the metabolomic signature.

The altered metabolomic profile in the models of disease was found to bear similarities with the altered profile observed in women who later developed preeclampsia or fetal growth restriction. These similarities serve to further validate these models as appropriate – not only for further investigation of potential mechanisms of disease, but also for use to test potential new treatments. As a paucity of appropriate animal models has been a factor in the development of curative treatments, this is an extremely important development and highlights the potential role of metabolomic profiling in developing new therapeutic strategies. Further, additional investigation of metabolites found to be altered in these models may provide information regarding both the pathophysiological mechanisms, and the potential mechanisms of action of therapeutic strategies.

## Materials and Methods

### Animals and treatments

All protocols were approved by the University of Alberta Health Sciences Animal Policy and Welfare Committee in accordance with the Canadian Council on Animal Care and conformed to the *Guide for the Care and Use of Laboratory Animals* (copyright 1996, National Academy of Science).

Pregnant mice were used in this study; female COMT^−/−^, Nos3^−/−^ and C57BL/6J mice (C57BL/5J mice are the background strain of both knockout models) of 8–12 weeks of age were used. They were mated nightly with males of a corresponding genotype. The morning a vaginal plug was detected was denoted as gestational day (GD) 0.5. A subgroup of COMT^−/−^ and C57BL/6J mice were treated with Sildenafil citrate; Sildenafil citrate (0.2 mg/ml in drinking water; equivalent to 300mg/day in a 70kg human, adjusted for altered pharmacokinetics in the mouse^56^) or normal drinking water was administered from GD12.5. Water intake between treated and untreated groups was compared and was not statistically different. Timing of drug administration was chosen to begin at the point when placental development is a) equivalent to the end of the first trimester in the human b) the uteroplacental circulation is open and is the main determinant of fetal growth^57^. Treatment continued to GD 18.5 (term is GD 19.5). A terminal blood sample was collected, from all groups of mice, via cardiac puncture at GD18.5. Blood was allowed to clot on ice, then serum prepared by centrifugation (samples were spun for 5 minutes, at 5000 RPM at a temperature of 4 °C). Serum samples were aliquoted, frozen in liquid nitrogen and stored at −80 °C until required. In total, samples from 23 C57BL/6J, 13 COMT^−/−^ and 13 Nos3^−/−^ mice were studied. In addition, samples from 10 C57BL/6J and 14 COMT^−/−^ mice treated with Sildenafil citrate were also studied.

### Metabolite separation and detection

A targeted quantitative metabolomics approach was applied to analyze serum samples using a commercially available direct flow injection and LC-MS/MS kit from BIOCRATES Life Sciences AG (Austria). This kit, in combination with an ABI 4000 Q-Trap (Applied Biosystems/MDS Sciex) mass spectrometer, can be used for the targeted identification and quantification of up to 180 different endogenous metabolites including amino acids, acylcarnitines, biogenic amines, glycerophospholipids, sphingolipids and sugars. The method used combines the derivatization and extraction of analytes, their separation via reverse-phase PLC and the selective mass-spectrometric detection using multiple reaction monitoring (MRM) pairs. Isotope-labeled internal standards and other internal standards were integrated in the kit plate filter for metabolite quantification. The Absolute*IDQ* kit includes a 96 deep-well plate with a filter plate attached with sealing tape, and reagents and solvents used to prepare the plate assay. The first 14 wells in the kit were used for calibration and quality control (one blank; three zero samples, seven standards and three quality control samples provided with each kit). All the serum samples were analyzed using the protocol described in the AbsoluteIDQ user manual. Briefly, serum samples, obtained from pregnant mice at the end of gestation, were thawed on ice, vortexed and then centrifuged at 13,000x g. 10 μL of each serum sample was loaded onto the center of the filter on the upper 96-well kit plate and dried in a stream of nitrogen. Subsequently, 20 μL of a 5% solution of phenyl-isothiocyanate was added for derivatization. After incubation, the filter spots were dried again using an evaporator. Extraction of metabolites was then achieved by adding 300 μL methanol containing 5 mM ammonium acetate. The extracts were obtained by centrifugation into the lower 96-deep well plate, followed by a dilution step with kit MS running solvent. Mass spectrometric analysis was performed on an API4000 Qtrap® tandem mass spectrometry instrument (Applied Biosystems/MDS Analytical Technologies, Foster City, CA) equipped with a solvent delivery system. The samples were delivered to the mass spectrometer using a reverse phase HPLC column (to analyze amino acids and biogenic amines) followed by a direct flow injection (DI) assay (to analyze acylcarnitines and lipids). The Biocrates MetIQ software was used to control the entire assay workflow, from sample registration to automated calculation of metabolite concentrations to the export of data into other data analysis programs. A targeted profiling scheme was used to quantitatively screen for known small molecule metabolites using multiple reaction monitoring, neutral loss and precursor ion scans.

### Statistical analysis

As the raw data for each measured metabolite from each of the groups (COMT^−/−^, Nos3^−/−^ and C57BL/6J mice, and then COMT^−/−^ and C57BL/6J mice following Sildenafil citrate treatment) were not normally distributed, the differences in metabolic profile distributions were evaluated, per metabolite, using the nonparametric Kruskal-Wallis one-way analysis of variance by ranks test, and for significant metabolites, pairwise group comparisons were performed using the Wilcoxon rank-sum test. Comparisons were considered statistically significant if p-values lower than 0.05 were obtained. Canonical Variate Analysis (CVA)^58^ was then performed using all metabolites whose serum concentrations were significantly changed (p < 0.05) in order to visualise the correlated multivariate discrimination between all 5 data groups. For the CVA analysis, data were log transformed, mean centred, and scaled to unit variance^59^. Missing values were imputed using the k-nearest neighbour method^60^. All of the statistical analyses were carried out using the Matlab scripting language (http://www.mathworks.com/). Initially, results from COMT^−/−^ and Nos3^−/−^ mice were compared with C57BL/6J mice, to determine what, if any, differences were present in the metabolomic signature of different models compared with that of their background strain. A comparison between the two models, COMT^−/−^ and Nos3^−/−^, was also made to determine if the differing pathophysiological mechanisms were reflected in changes in the altered metabolomic signature. COMT^−/−^ mice treated with Sildenafil were compared with untreated C57BL/6J mice to determine the ability of Sildenafil to normalize the metabolomic signature in comparison with ‘normal’ control mice; treated C57BL/6J mice were also included in the event that Sildenafil treatment may alter metabolite concentration in normal mice.

## Additional Information

**How to cite this article**: Stanley, J. L. *et al.* Sildenafil Therapy Normalizes the Aberrant Metabolomic Profile in the Comt^-/-^ Mouse Model of Preeclampsia / Fetal Growth Restriction. *Sci. Rep.* **5**, 18241; doi: 10.1038/srep18241 (2015).

## Supplementary Material

Supplementary Information

## Figures and Tables

**Table 1:** Serum metabolomic signature of COMT^−/−^ mice with and without Sildenafil citrate treatment compared with control C57BL/6J mice.

***Class***	***Name***	***Metabolites concentrations [uM]***		
		***COMT***	***C57***	***COMT Sc***
Acylcarnitine	C2	14.88 (12.83–19.06)*	22.04 (18.17–26.48)	20.87 (16.50–24.39)
	C3	0.66 (0.48–0.72)**	0.93 (0.71–1.13)	0.85 (0.66–0.95)
	C4	0.53 (0.0.46–0.63)**	1.02 (0.76–1.13)	0.66 (0.56–0.77)*
	C5	0.08 (0.07–0.12)*	0.13 (0.10–0.17)	0.12 (0.11–0.16)
	C6 (C4:1–DC)	0.09 (0.07–0.12)**	0.13 (0.10–0.17)	0.10 (0.08–0.12)
Glycerophospholipids	PC aa C30:0	0.52 (0.51–0.59)**	0.47 (0.46–0.51)	0.57 (0.49–0.60)*
	PC aa C32:3	0.11 (0.10–0.13)*	0.09 (0.07–0.11)	0.10 (0.08–0.10)
	PC aa C34:2	176.95 (147.77–195.22)**	145.53 (138.19–155.25)	141.40 (129.82–146.94)
	PC aa C36:0	1.19 (1.12–1.27)*	1.09 (0.90–1.14)	1.21 (1.10–1.33)
	PC aa C36:5	6.93 (6.23–8.03)**	5.45 (4.92–6.17)	5.89 (4.77–6.81)
	PC aa C38:0	1.64 (1.48–1.79)*	1.36 (1.29–1.50)	1.52 (1.45–1.86)*
	PC aa C42:0	0.10 (0.08–0.13)*	0.08 (0.06–0.10)	0.09 (0.08–0.11)
	PC ae C32:2	0.17 (0.15–0.20)*	0.14 (0.13–0.16)	0.16 (0.14–0.19)
	PC ae C34:2	1.74 (1.53–1.94)***	1.31 (1.21–1.43)	1.47 (1.36–1.54)*
	PC ae C34:3	0.41 (0.35–0.49)*	0.31 (0.28–0.36)	0.40 (0.34–0.45)**
	PC ae C36:2	3.95 (3.61–4.37)*	3.26 (3.10–3.58)	3.35 (3.24–3.54)
	PC ae C36:3	0.92 (0.80–1.03)**	0.73 (0.67–0.82)	0.79 (0.78–0.85)
	lysoPC a C14:0	3.44 (2.42–4.66)*	4.51 (3.97–5.12)	5.15 (4.19–6.03)
Sphingolipids	SM C16:0	24.34 (22.87–24.91)***	19.98 (17.17–21.81)	19.90 (19.00–26.79)
	SM C16:1	2.06 (1.80–2.47)*	1.75 (1.51–1.92)	1.83 (1.64–2.23)
	SM C24:1	16.11 (13.93–18.68)*	12.87 (10.87–15.03)	12.81 (11.88–17.94)
Biogenic amines	Kynurenine	2.83 (1.90–2.69)**	3.49 (2.48–3.92)	2.86 (2.36–3.76)

Data presented as median (interquartile range); Statistical differences calculated between either COMT^−/−^ (COMT) vs. control C57BL/6J (C57) mice or COMT^−/−^ mice treated with Sildenafil citrate (COMT Sc) vs. control C57BL/6J mice; *p < 0.05; **p < 0.01; ***p < 0.001.

## References

[b1] Knight M. Eclampsia in the United Kingdom 2005. BJOG 114, 1072–1078, 10.1111/j.1471-0528.2007.01423.x (2007). 17617191

[b2] Lewis G. The Confidential Enquiry into Maternal and Child Health (CEMACH). Saving Mothers’ Lives: Reviewing maternal deaths to make motherhood safer - 2003–2005. The seventh report on confidential enquiries into maternal deaths in the United Kingdom (CEMACH, London, 2007).

[b3] Kuklina E. V., Ayala C. & Callaghan W. M. Hypertensive disorders and severe obstetric morbidity in the United States. Obstet Gynecol 113, 1299–1306, 10.1097/AOG.0b013e3181a45b25 (2009). 19461426

[b4] Cantwell R. *et al.* Saving Mothers’ Lives: Reviewing maternal deaths to make motherhood safer: 2006–2008. The Eighth Report of the Confidential Enquiries into Maternal Deaths in the United Kingdom. *BJOG* 118 Suppl 1, 1–203, doi: 10.1111/j.1471-0528.2010.02847.x (2011). 10.1111/j.1471-0528.2010.02847.x 21356004

[b5] Gardosi J., Madurasinghe V., Williams M., Malik A. & Francis A. Maternal and fetal risk factors for stillbirth: population based study. BMJ 346, f108, 10.1136/bmj.f108 (2013). 23349424 PMC3554866

[b6] Barker D. J. Fetal growth and adult disease. Br J Obstet Gynaecol 99, 275–276 (1992). 1581269 10.1111/j.1471-0528.1992.tb13719.x

[b7] Lithell H. O. *et al.* Relation of size at birth to non-insulin dependent diabetes and insulin concentrations in men aged 50–60 years. BMJ 312, 406–410 (1996). 8601111 10.1136/bmj.312.7028.406 PMC2350082

[b8] Harrington K., Cooper D., Lees C., Hecher K. & Campbell S. Doppler ultrasound of the uterine arteries: the importance of bilateral notching in the prediction of pre-eclampsia, placental abruption or delivery of a small-for-gestational-age baby. Ultrasound Obstet Gynecol 7, 182–188, 10.1046/j.1469-0705.1996.07030182.x (1996). 8705410

[b9] Myatt L., Brewer A. S., Langdon G. & Brockman D. E. Attenuation of the vasoconstrictor effects of thromboxane and endothelin by nitric oxide in the human fetal-placental circulation. Am J Obstet Gynecol 166, 224–230 (1992). 1733199 10.1016/0002-9378(92)91863-6

[b10] Nelson S. H. *et al.* Increased nitric oxide synthase activity and expression in the human uterine artery during pregnancy. Circ Res 87, 406–411 (2000). 10969039 10.1161/01.res.87.5.406

[b11] Williams D. J., Vallance P. J., Neild G. H., Spencer J. A. & Imms F. J. Nitric oxide-mediated vasodilation in human pregnancy. Am J Physiol 272, H748–752 (1997). 9124434 10.1152/ajpheart.1997.272.2.H748

[b12] van der Heijden O. W. *et al.* Uterine artery remodeling and reproductive performance are impaired in endothelial nitric oxide synthase-deficient mice. Biol Reprod 72, 1161–1168, 10.1095/biolreprod.104.033985 (2005). 15659709

[b13] Kusinski L. C. *et al.* eNOS knockout mouse as a model of fetal growth restriction with an impaired uterine artery function and placental transport phenotype. Am J Physiol Regul Integr Comp Physiol 303, R86–93, 10.1152/ajpregu.00600.2011 (2012). 22552791

[b14] Kulandavelu S., Whiteley K. J., Bainbridge S. A., Qu D. & Adamson S. L. Endothelial NO synthase augments fetoplacental blood flow, placental vascularization, and fetal growth in mice. Hypertension 61, 259–266, 10.1161/HYPERTENSIONAHA.112.201996 (2013). 23150513

[b15] Stanley J. L. *et al.* Effect of the Anti-Oxidant Tempol on Fetal Growth in a Mouse Model of Fetal Growth Restriction. Biol Reprod, 10.1095/biolreprod.111.096198 (2012). PMC3406559 22423051

[b16] Zhu B. T. & Conney A. H. Functional role of estrogen metabolism in target cells: review and perspectives. Carcinogenesis 19, 1–27 (1998). 9472688 10.1093/carcin/19.1.1

[b17] Kanasaki K. *et al.* Deficiency in catechol-O-methyltransferase and 2-methoxyoestradiol is associated with pre-eclampsia. Nature 453, 1117–1121, 10.1038/nature06951 (2008). 18469803

[b18] Stanley J. L. *et al.* Sildenafil Citrate Rescues Fetal Growth in the Catechol-O-Methyl Transferase Knockout Mouse Model. Hypertension, 10.1161/HYPERTENSIONAHA.111.186270 (2012). 22392899

[b19] von Dadelszen P. *et al.* Sildenafil citrate therapy for severe early-onset intrauterine growth restriction. BJOG 118, 624–628, 10.1111/j.1471-0528.2010.02879.x (2011). 21392225

[b20] Campbell S. *et al.* New doppler technique for assessing uteroplacental blood flow. Lancet 1, 675–677, 10.1016/S0140-6736(83)92546-1 (1983). 6132039

[b21] Gilbert J. S., Babcock S. A. & Granger J. P. Hypertension produced by reduced uterine perfusion in pregnant rats is associated with increased soluble fms-like tyrosine kinase-1 expression. Hypertension 50, 1142–1147, 10.1161/HYPERTENSIONAHA.107.096594 (2007). 17923588

[b22] Ramirez R. J., Debrah J. & Novak J. Increased myogenic responses of resistance-sized mesenteric arteries after reduced uterine perfusion pressure in pregnant rats. Hypertens Pregnancy 30, 45–57, 10.3109/10641950903322923 (2011). 20818955

[b23] Morton J. S. *et al.* Lectin-like oxidized low-density lipoprotein 1 receptor in a reduced uteroplacental perfusion pressure rat model of preeclampsia. Hypertension 59, 1014–1020, 10.1161/HYPERTENSIONAHA.112.191825 (2012). 22392901

[b24] Woodall S. M., Breier B. H., Johnston B. M. & Gluckman P. D. A model of intrauterine growth retardation caused by chronic maternal undernutrition in the rat: effects on the somatotrophic axis and postnatal growth. J Endocrinol 150, 231–242 (1996). 8869590 10.1677/joe.0.1500231

[b25] Resnick O., Morgane P. J., Hasson R. & Miller M. Overt and hidden forms of chronic malnutrition in the rat and their relevance to man. Neurosci Biobehav Rev 6, 55–75, 10.1016/0149-7634(82)90007-0 (1982). 6803197

[b26] Hefler L. A., Tempfer C. B., Moreno R. M., O’Brien W. E. & Gregg A. R. Endothelial-derived nitric oxide and angiotensinogen: blood pressure and metabolism during mouse pregnancy. Am J Physiol Regul Integr Comp Physiol 280, R174–182 (2001). 11124149 10.1152/ajpregu.2001.280.1.R174

[b27] Huang P. L. *et al.* Hypertension in mice lacking the gene for endothelial nitric oxide synthase. Nature 377, 239–242, 10.1038/377239a0 (1995). 7545787

[b28] Chataigneau T. *et al.* Acetylcholine-induced relaxation in blood vessels from endothelial nitric oxide synthase knockout mice. Br J Pharmacol 126, 219–226, 10.1038/sj.bjp.0702300 (1999). 10051139 PMC1565804

[b29] Kulandavelu S., Qu D. & Adamson S. L. Cardiovascular function in mice during normal pregnancy and in the absence of endothelial NO synthase. Hypertension 47, 1175–1182, 10.1161/01.HYP.0000218440.71846.db (2006). 16636199

[b30] Kulandavelu S. *et al.* Endothelial Nitric Oxide Synthase Deficiency Reduces Uterine Blood Flow, Spiral Artery Elongation, and Placental Oxygenation in Pregnant Mice. Hypertension 60, 231–238, 10.1161/Hypertensionaha.111.187559 (2012). 22615111

[b31] English F. A. *et al.* Administration of the PARP inhibitor Pj34 ameliorates the impaired vascular function associated with eNOS(^−/−^) mice. Reprod Sci 19, 806–813, 10.1177/1933719111433885 (2012). 22421449

[b32] Gulcin I. Antioxidant and antiradical activities of L-carnitine. Life Sci 78, 803–811, 10.1016/j.lfs.2005.05.103 (2006). 16253281

[b33] Aydin A. F., Kucukgergin C., Ozdemirler-Erata G., Kocak-Toker N. & Uysal M. The effect of carnosine treatment on prooxidant-antioxidant balance in liver, heart and brain tissues of male aged rats. Biogerontology 11, 103–109, 10.1007/s10522-009-9232-4 (2010). 19430956

[b34] Gogos J. A. *et al.* Catechol-O-methyltransferase-deficient mice exhibit sexually dimorphic changes in catecholamine levels and behavior. Proc Natl Acad Sci USA 95, 9991–9996 (1998). 9707588 10.1073/pnas.95.17.9991 PMC21449

[b35] Berg D., Sonsalla R. & Kuss E. Concentrations of 2-Methoxyestrogens in Human-Serum Measured by a Heterologous Immunoassay with an I-125-Labeled Ligand. Acta Endocrinol-Cop 103, 282–288 (1983). 10.1530/acta.0.1030282 6858558

[b36] Bartlett K. & Eaton S. Mitochondrial beta-oxidation. Eur J Biochem 271, 462–469, 10.1046/j.1432-1033.2003.03947.x (2004). 14728673

[b37] Hoppel C. L. & Genuth S. M. Carnitine metabolism in normal-weight and obese human subjects during fasting. Am J Physiol 238, E409–415 (1980). 7377339 10.1152/ajpendo.1980.238.5.E409

[b38] Adams S. H. *et al.* Plasma acylcarnitine profiles suggest incomplete long-chain fatty acid beta-oxidation and altered tricarboxylic acid cycle activity in type 2 diabetic African-American women. J Nutr 139, 1073–1081, 10.3945/jn.108.103754 (2009). 19369366 PMC2714383

[b39] Koves T. R. *et al.* Peroxisome proliferator-activated receptor-gamma co-activator 1alpha-mediated metabolic remodeling of skeletal myocytes mimics exercise training and reverses lipid-induced mitochondrial inefficiency. J Biol Chem 280, 33588–33598, 10.1074/jbc.M507621200 (2005). 16079133

[b40] Madeleneau D. *et al.* Transcriptomic analysis of human placenta in intrauterine growth restriction. Pediatr Res 77, 799–807, 10.1038/pr.2015.40 (2015). 25734244

[b41] Mayeur S. *et al.* Maternal calorie restriction modulates placental mitochondrial biogenesis and bioenergetic efficiency: putative involvement in fetoplacental growth defects in rats. Am J Physiol Endocrinol Metab 304, E14–22, 10.1152/ajpendo.00332.2012 (2013). 23092912

[b42] Mando C. *et al.* Placental mitochondrial content and function in intrauterine growth restriction and preeclampsia. Am J Physiol Endocrinol Metab 306, E404–413, 10.1152/ajpendo.00426.2013 (2014). 24347055

[b43] Nisoli E. *et al.* Mitochondrial biogenesis by NO yields functionally active mitochondria in mammals. Proc Natl Acad Sci USA 101, 16507–16512, 10.1073/pnas.0405432101 (2004). 15545607 PMC534517

[b44] Whitaker R. M., Wills L. P., Stallons L. J. & Schnellmann R. G. cGMP-selective phosphodiesterase inhibitors stimulate mitochondrial biogenesis and promote recovery from acute kidney injury. J Pharmacol Exp Ther 347, 626–634, 10.1124/jpet.113.208017 (2013). 24042162 PMC3836317

[b45] Mitschke M. M. *et al.* Increased cGMP promotes healthy expansion and browning of white adipose tissue. FASEB J 27, 1621–1630, 10.1096/fj.12-221580 (2013). 23303211

[b46] Creider J. C., Hegele R. A. & Joy T. R. Niacin: another look at an underutilized lipid-lowering medication. Nat Rev Endocrinol 8, 517–528, 10.1038/nrendo.2012.22 (2012). 22349076

[b47] Wu B. J. *et al.* Evidence that niacin inhibits acute vascular inflammation and improves endothelial dysfunction independent of changes in plasma lipids. Arterioscler Thromb Vasc Biol 30, 968–975, 10.1161/ATVBAHA.109.201129 (2010). 20167660

[b48] Pereto J., Lopez-Garcia P. & Moreira D. Ancestral lipid biosynthesis and early membrane evolution. Trends Biochem Sci 29, 469–477, 10.1016/j.tibs.2004.07.002 (2004). 15337120

[b49] Melland-Smith M. *et al.* Disruption of sphingolipid metabolism augments ceramide-induced autophagy in preeclampsia. Autophagy 11, 653–669, 10.1080/15548627.2015.1034414 (2015). 25853898 PMC4502662

[b50] De Nadai C. *et al.* Nitric oxide inhibits tumor necrosis factor-alpha-induced apoptosis by reducing the generation of ceramide. Proc Natl Acad Sci USA 97, 5480–5485, 10.1073/pnas.070062397 (2000). 10792026 PMC25854

[b51] Heazell A. E., Buttle H. R., Baker P. N. & Crocker I. P. Altered expression of regulators of caspase activity within trophoblast of normal pregnancies and pregnancies complicated by preeclampsia. Reprod Sci 15, 1034–1043, 10.1177/1933719108322438 (2008). 19088373

[b52] Smith S. C., Baker P. N. & Symonds E. M. Increased placental apoptosis in intrauterine growth restriction. Am J Obstet Gynecol 177, 1395–1401, 10.1016/S0002-9378(97)70081-4 (1997). 9423741

[b53] Horgan R. P. *et al.* Metabolic profiling uncovers a phenotypic signature of small for gestational age in early pregnancy. J Proteome Res 10, 3660–3673, 10.1021/pr2002897 (2011). 21671558

[b54] Kenny L. C. *et al.* Robust early pregnancy prediction of later preeclampsia using metabolomic biomarkers. Hypertension 56, 741–749, 10.1161/HYPERTENSIONAHA.110.157297 (2010). 20837882 PMC7614124

[b55] Meng T. *et al.* Identification of differential gene expression profiles in placentas from preeclamptic pregnancies versus normal pregnancies by DNA microarrays. OMICS 16, 301–311, 10.1089/omi.2011.0066 (2012). 22702245 PMC3369279

[b56] Walker D. K. *et al.* Pharmacokinetics and metabolism of sildenafil in mouse, rat, rabbit, dog and man. Xenobiotica 29, 297–310, 10.1080/004982599238687 (1999). 10219969

[b57] Sapin V. *et al.* Use of transgenic mice model for understanding the placentation: towards clinical applications in human obstetrical pathologies? Transgenic Res 10, 377–398 (2001). 11708649 10.1023/a:1012085713898

[b58] Krzanowski W. Principles of Multivariate Analysis: A User’s Perspective. (Oxfore University Press, 1988).

[b59] van den Berg R. A., Hoefsloot H. C., Westerhuis J. A., Smilde A. K. & van der Werf M. J. Centering, scaling, and transformations: improving the biological information content of metabolomics data. BMC Genomics 7, 142, 10.1186/1471-2164-7-142 (2006). 16762068 PMC1534033

[b60] Speed T. P. Statistical analysis of gene expression microarray data. (Chapman & Hall/CRC, 2003).

