# Supplementary material for: Sildenafil Therapy Normalizes the Aberrant Metabolomic Profile in the Comt−/− Mouse Model of Preeclampsia/Fetal Growth Restriction
Source: Sci Rep. 2015 Dec 15;5:18241. doi: 10.1038/srep18241 (PMC4678899; doi:10.1038/srep18241)
Supplement: Supplementary Information [file srep18241-s1.doc]

**SILDENAFIL THERAPY NORMALIZES THE ABERRANT METABOLOMIC PROFILE IN THE COMT^-/-^ MOUSE MODEL OF PREECLAMPSIA / FETAL GROWTH RESTRICTION**

Joanna L Stanley^1,2^* & Karolina Sulek^1^, Irene J Andersson^2^, Sandra T Davidge^3^, Louise C Kenny^4^, Colin P Sibley^5^, Rupasri Mandal^6^, David S Wishart^6^, David I Broadhurst^2^ and Philip N Baker^1^

^1^Liggins Institute, University of Auckland, New Zealand

^2^Department of Medicine, University of Alberta, Edmonton, Canada

^3^Women and Children’s Health Research Institute, University of Alberta, Edmonton, Canada

^4^Anu Research Centre, University College Cork, Ireland

^5^Maternal & Fetal Health Research Centre, Institute of Human Development, Faculty of Medical and Human Sciences, University of Manchester, U.K.

^6^The Metabolomics Innovation Centre (TMIC), University of Alberta, Edmonton, Canada

* Corresponding author: Dr J L Stanley, Liggins Institute, 85 Park Road, Grafton, Auckland, New Zealand. 1023

Ph:+64 9 923 4616, fax:+64 9 373 7039, j.stanley@auckland.ac.nz

**Online Data Supplement**

**Results**

**Table S1.** Full names and abbreviations of metabolites identified by LC-MS/MS

| ***Abbreviation*** | ***Full Name*** | ***Class*** |
| --- | --- | --- |
| C0 | Carnitine | Acylcarnitines |
| C10:1 | Decenoylcarnitine | Acylcarnitines |
| C12 | Dodecanoylcarnitine | Acylcarnitines |
| C14 | Tetradecanoylcarnitine | Acylcarnitines |
| C14:1 | Tetradecenoylcarnitine | Acylcarnitines |
| C14:1-OH | Hydroxytetradecenoylcarnitine | Acylcarnitines |
| C14:2 | tetradecadienylcarnitine | Acylcarnitines |
| C16 | Hexadecanoylcarnitine | Acylcarnitines |
| C16-OH | Hydroxyhexadecanoylcarnitine | Acylcarnitines |
| C16:1 | Hexadecenoylcarnitine | Acylcarnitines |
| C16:1-OH | Hydroxyhexadecenoylcarnitine | Acylcarnitines |
| C16:2 | Hexadecadienylcarnitine | Acylcarnitines |
| C18 | Octadecanoylcarnitine | Acylcarnitines |
| C18:1 | Octadecenoylcarnitine | Acylcarnitines |
| C18:1-OH | Hydroxyoctadecenoylcarnitine | Acylcarnitines |
| C18:2 | Octadecadienylcarnitine | Acylcarnitines |
| C2 | Acetylcarnitine | Acylcarnitines |
| C3 | Propionylcarnitine | Acylcarnitines |
| C3-DC (C4-OH) | Hydroxybutyrylcarnitine | Acylcarnitines |
| C4 | Butyrylcarnitine | Acylcarnitines |
| C5 | Valerylcarnitine | Acylcarnitines |
| C6 (C4:1-DC) | Hexanoylcarnitine | Acylcarnitines |
| C9 | Nonaylcarnitine | Acylcarnitines |
| **PC aa** C24:0 | **Phosphatidylcholine diacyl** C24:0 | Glycerophospholipids |
| PC aa C28:1 | Phosphatidylcholine diacyl C28:1 | Glycerophospholipids |
| PC aa C30:0 | Phosphatidylcholine diacyl C30:0 | Glycerophospholipids |
| PC aa C32:0 | Phosphatidylcholine diacyl C32:0 | Glycerophospholipids |
| PC aa C32:1 | Phosphatidylcholine diacyl C32:1 | Glycerophospholipids |
| PC aa C32:2 | Phosphatidylcholine diacyl C32:2 | Glycerophospholipids |
| PC aa C32:3 | Phosphatidylcholine diacyl C32:3 | Glycerophospholipids |
| PC aa C34:1 | Phosphatidylcholine diacyl C34:1 | Glycerophospholipids |
| PC aa C34:2 | Phosphatidylcholine diacyl C34:2 | Glycerophospholipids |
| PC aa C34:3 | Phosphatidylcholine diacyl C34:3 | Glycerophospholipids |
| PC aa C34:4 | Phosphatidylcholine diacyl C34:4 | Glycerophospholipids |
| PC aa C36:0 | Phosphatidylcholine diacyl C36:0 | Glycerophospholipids |
| PC aa C36:1 | Phosphatidylcholine diacyl C36:1 | Glycerophospholipids |
| PC aa C36:2 | Phosphatidylcholine diacyl C36:2 | Glycerophospholipids |
| PC aa C36:3 | Phosphatidylcholine diacyl C36:3 | Glycerophospholipids |
| PC aa C36:4 | Phosphatidylcholine diacyl C36:4 | Glycerophospholipids |
| PC aa C36:5 | Phosphatidylcholine diacyl C36:5 | Glycerophospholipids |
| PC aa C36:6 | Phosphatidylcholine diacyl C36:6 | Glycerophospholipids |
| PC aa C38:0 | Phosphatidylcholine diacyl C38:0 | Glycerophospholipids |
| PC aa C38:1 | Phosphatidylcholine diacyl C38:1 | Glycerophospholipids |
| PC aa C38:3 | Phosphatidylcholine diacyl C38:3 | Glycerophospholipids |
| PC aa C38:4 | Phosphatidylcholine diacyl C38:4 | Glycerophospholipids |
| PC aa C38:5 | Phosphatidylcholine diacyl C38:5 | Glycerophospholipids |
| PC aa C38:6 | Phosphatidylcholine diacyl C38:6 | Glycerophospholipids |
| PC aa C40:2 | Phosphatidylcholine diacyl C40:2 | Glycerophospholipids |
| PC aa C40:3 | Phosphatidylcholine diacyl C40:3 | Glycerophospholipids |
| PC aa C40:4 | Phosphatidylcholine diacyl C40:4 | Glycerophospholipids |
| PC aa C40:5 | Phosphatidylcholine diacyl C40:5 | Glycerophospholipids |
| PC aa C40:6 | Phosphatidylcholine diacyl C40:6 | Glycerophospholipids |
| PC aa C42:0 | Phosphatidylcholine diacyl C42:0 | Glycerophospholipids |
| PC aa C42:1 | Phosphatidylcholine diacyl C42:1 | Glycerophospholipids |
| PC aa C42:2 | Phosphatidylcholine diacyl C42:2 | Glycerophospholipids |
| PC aa C42:4 | Phosphatidylcholine diacyl C42:4 | Glycerophospholipids |
| PC aa C42:5 | Phosphatidylcholine diacyl C42:5 | Glycerophospholipids |
| PC aa C42:6 | Phosphatidylcholine diacyl C42:6 | Glycerophospholipids |
| **PC ae** C30:0 | **Phosphatidylcholine acyl-alkyl** C30:0 | Glycerophospholipids |
| PC ae C30:1 | Phosphatidylcholine acyl-alkyl C30:1 | Glycerophospholipids |
| PC ae C32:1 | Phosphatidylcholine acyl-alkyl C32:1 | Glycerophospholipids |
| PC ae C32:2 | Phosphatidylcholine acyl-alkyl C32:2 | Glycerophospholipids |
| PC ae C34:0 | Phosphatidylcholine acyl-alkyl C34:0 | Glycerophospholipids |
| PC ae C34:1 | Phosphatidylcholine acyl-alkyl C34:1 | Glycerophospholipids |
| PC ae C34:2 | Phosphatidylcholine acyl-alkyl C34:2 | Glycerophospholipids |
| PC ae C34:3 | Phosphatidylcholine acyl-alkyl C34:3 | Glycerophospholipids |
| PC ae C36:0 | Phosphatidylcholine acyl-alkyl C36:0 | Glycerophospholipids |
| PC ae C36:1 | Phosphatidylcholine acyl-alkyl C36:1 | Glycerophospholipids |
| PC ae C36:2 | Phosphatidylcholine acyl-alkyl C36:2 | Glycerophospholipids |
| PC ae C36:3 | Phosphatidylcholine acyl-alkyl C36:3 | Glycerophospholipids |
| PC ae C36:4 | Phosphatidylcholine acyl-alkyl C36:4 | Glycerophospholipids |
| PC ae C36:5 | Phosphatidylcholine acyl-alkyl C36:5 | Glycerophospholipids |
| PC ae C38:0 | Phosphatidylcholine acyl-alkyl C38:0 | Glycerophospholipids |
| PC ae C38:1 | Phosphatidylcholine acyl-alkyl C38:1 | Glycerophospholipids |
| PC ae C38:2 | Phosphatidylcholine acyl-alkyl C38:2 | Glycerophospholipids |
| PC ae C38:3 | Phosphatidylcholine acyl-alkyl C38:3 | Glycerophospholipids |
| PC ae C38:4 | Phosphatidylcholine acyl-alkyl C38:4 | Glycerophospholipids |
| PC ae C38:5 | Phosphatidylcholine acyl-alkyl C38:5 | Glycerophospholipids |
| PC ae C38:6 | Phosphatidylcholine acyl-alkyl C38:6 | Glycerophospholipids |
| PC ae C40:1 | Phosphatidylcholine acyl-alkyl C40:1 | Glycerophospholipids |
| PC ae C40:2 | Phosphatidylcholine acyl-alkyl C40:2 | Glycerophospholipids |
| PC ae C40:3 | Phosphatidylcholine acyl-alkyl C40:3 | Glycerophospholipids |
| PC ae C40:4 | Phosphatidylcholine acyl-alkyl C40:4 | Glycerophospholipids |
| PC ae C40:5 | Phosphatidylcholine acyl-alkyl C40:5 | Glycerophospholipids |
| PC ae C40:6 | Phosphatidylcholine acyl-alkyl C40:6 | Glycerophospholipids |
| PC ae C42:0 | Phosphatidylcholine acyl-alkyl C42:0 | Glycerophospholipids |
| PC ae C42:1 | Phosphatidylcholine acyl-alkyl C42:1 | Glycerophospholipids |
| PC ae C42:2 | Phosphatidylcholine acyl-alkyl C42:2 | Glycerophospholipids |
| PC ae C42:3 | Phosphatidylcholine acyl-alkyl C42:3 | Glycerophospholipids |
| PC ae C44:3 | Phosphatidylcholine acyl-alkyl C44:3 | Glycerophospholipids |
| PC ae C44:4 | Phosphatidylcholine acyl-alkyl C44:4 | Glycerophospholipids |
| PC ae C44:5 | Phosphatidylcholine acyl-alkyl C44:5 | Glycerophospholipids |
| **lysoPC a** C14:0 | **lysoPhosphatidylcholine acyl** C14:0 | Glycerophospholipids |
| lysoPC a C16:0 | lysoPhosphatidylcholine acyl C16:0 | Glycerophospholipids |
| lysoPC a C16:1 | lysoPhosphatidylcholine acyl C16:1 | Glycerophospholipids |
| lysoPC a C17:0 | lysoPhosphatidylcholine acyl C17:0 | Glycerophospholipids |
| lysoPC a C18:0 | lysoPhosphatidylcholine acyl C18:0 | Glycerophospholipids |
| lysoPC a C18:1 | lysoPhosphatidylcholine acyl C18:1 | Glycerophospholipids |
| lysoPC a C18:2 | lysoPhosphatidylcholine acyl C18:2 | Glycerophospholipids |
| lysoPC a C20:3 | lysoPhosphatidylcholine acyl C20:3 | Glycerophospholipids |
| lysoPC a C20:4 | lysoPhosphatidylcholine acyl C20:4 | Glycerophospholipids |
| lysoPC a C26:0 | lysoPhosphatidylcholine acyl C26:0 | Glycerophospholipids |
| lysoPC a C28:0 | lysoPhosphatidylcholine acyl C28:0 | Glycerophospholipids |
| lysoPC a C28:1 | lysoPhosphatidylcholine acyl C28:1 | Glycerophospholipids |
| **SM (OH)** C14:1 | **Hydroxysphingomyeline** C14:1 | Sphingolipids |
| SM (OH) C16:1 | Hydroxysphingomyeline C16:1 | Sphingolipids |
| SM (OH) C22:1 | Hydroxysphingomyeline C22:1 | Sphingolipids |
| SM (OH) C22:2 | Hydroxysphingomyeline C22:2 | Sphingolipids |
| SM (OH) C24:1 | Hydroxysphingomyeline C24:1 | Sphingolipids |
| **SM** C16:0 | **Sphingomyeline** C16:0 | Sphingolipids |
| SM C16:1 | Sphingomyeline C16:1 | Sphingolipids |
| SM C18:0 | Sphingomyeline C18:0 | Sphingolipids |
| SM C18:1 | Sphingomyeline C18:1 | Sphingolipids |
| SM C20:2 | Sphingomyeline C20:2 | Sphingolipids |
| SM C22:3 | Sphingomyeline C22:3 | Sphingolipids |
| SM C24:0 | Sphingomyeline C24:0 | Sphingolipids |
| SM C24:1 | Sphingomyeline C24:1 | Sphingolipids |
| SM C26:0 | Sphingomyeline C26:0 | Sphingolipids |
| SM C26:1 | Sphingomyeline C26:1 | Sphingolipids |
| Hexose | Hexose | Sugars |
| Alanine | Alanine | Amino acids |
| Arginine | Arginine | Amino acids |
| Asparagine | Asparagine | Amino acids |
| Citrulline | Citrulline | Amino acids |
| Glutamine | Glutamine | Amino acids |
| Glycine | Glycine | Amino acids |
| Histidine | Histidine | Amino acids |
| Isoleucine | Isoleucine | Amino acids |
| Leucine | Leucine | Amino acids |
| Lysine | Lysine | Amino acids |
| Methionine | Methionine | Amino acids |
| Ornithine | Ornithine | Amino acids |
| Phenylalanine | Phenylalanine | Amino acids |
| Proline | Proline | Amino acids |
| Serine | Serine | Amino acids |
| Threonine | Threonine | Amino acids |
| Tryptophan | Tryptophan | Amino acids |
| Tyrosine | Tyrosine | Amino acids |
| Valine | Valine | Amino acids |
| Ac-Orn | Acetyl ornithine | Biogenic amines |
| ADMA | Asymmetric dimethylarginine | Biogenic amines |
| α-AAA | α-Aminoadipic acid | Biogenic amines |
| Carnosine | Carnosine | Biogenic amines |
| Creatinine | Creatinine | Biogenic amines |
| Histamine | Histamine | Biogenic amines |
| Kynurenine | Kynurenine | Biogenic amines |
| Met-SO | Methionine-S-Sulfoxide | Biogenic amines |
| Sarcosine | Sarcosine | Biogenic amines |
| Spermidine | Spermidine | Biogenic amines |


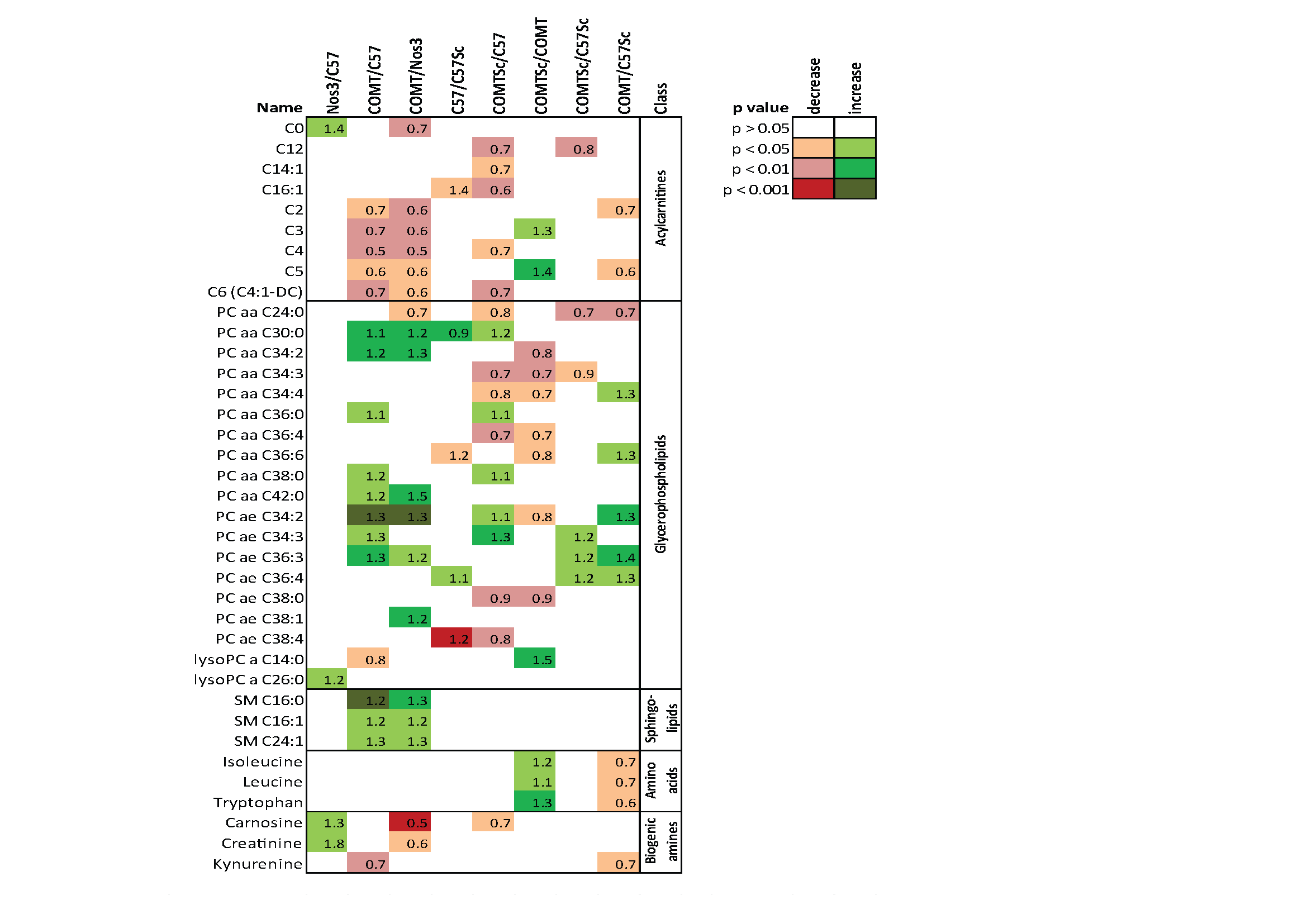


**Figure S1.** Heat map representing statistical differences, assessed by Kruskal-Wallis test, between Nos3^-/-^, COMT^-/-^ and C57BL/6J mice, as well as those treated with Sildenafil citrate. Legend shows color-coding for statistical differences in the concentrations of given metabolites. Numbers in the marked fields represent ratios between the medians of given groups.
